# Triggering The Birth of New Cycle’s Sunspots by Solar Tsunami

**DOI:** 10.1038/s41598-018-37939-z

**Published:** 2019-02-14

**Authors:** Mausumi Dikpati, Scott W. McIntosh, Subhamoy Chatterjee, Dipankar Banerjee, Ron Yellin-Bergovoy, Abhishek Srivastava

**Affiliations:** 1 High Altitude Observatory, National Center for Atmospheric Research, 3080 Center Green, Boulder, CO 80301 USA; 2 Indian Institute of Astrophysics, Koramangala, Bangalore, 560034 India; 3 Department of Geosciences, Tel-Aviv University, Tel-Aviv, Israel; 4 Department of Physics, Indian Institute of Technology (BHU), Varanasi, 221005 India

## Abstract

When will a new cycle’s sunspots appear? We demonstrate a novel physical mechanism, namely, that a “solar tsunami” occurring in the Sun’s interior shear-fluid layer can trigger new cycle’s magnetic flux emergence at high latitudes, a few weeks after the cessation of old cycle’s flux emergence near the equator. This tsunami is excited at the equator when magnetic dams, created by the oppositely-directed old cycle’s toroidal field in North and South hemispheres, break due to mutual annihilation of toroidal flux there. The fluid supported by these dams rushes to the equator; the surplus of fluid cannot be contained there, so it reflects back towards high latitudes, causing a tsunami. This tsunami propagates poleward at a speed of ~300 m/s until it encounters the new cycle’s spot-producing toroidal fields in mid-latitudes, where it perturbs the fields, triggering their surface-eruption in the form of new cycle spots. A new sunspot cycle is preceded for several years by other forms of high-latitude magnetic activity, such as coronal bright points and ephemeral regions, until the tsunami causes the birth of new cycle’s spots. The next tsunami is due by 2020, portending the start of intense ‘space weather’ that can adversely impact the Earth.

## Introduction

On the verge of every solar activity minimum we wait to see when the Sun will change its “mood” from quiet phase to sudden activity bursts, which we know will lead soon to major events of space weather. It will be very valuable to predict in advance precisely when this mood change will occur. Are there “precursor events” that would allow us to predict the time of birth of new cycle’s sunspots to an accuracy of a few weeks?

There is increasing evidence that the appearance of a new sunspot cycle is preceded by high-latitude magnetic events in various forms, including coronal bright points, polar crown filaments, ephemeral magnetic regions, which is called the ‘extended solar cycle’. The concept of an ‘extended’ cycle was first presented by Wilson^1^ *et al*. in 1988. Evidence supporting this concept originally was the occurrence of new ‘ephemeral’ active regions at higher latitudes than spots are seen, several years before new cycle spots were observed at lower latitudes. Subsequently other high latitude magnetic activity signatures were added to this picture, and the torsional oscillations of the Sun’s differential rotation were also found to extend poleward of sunspot latitudes. Cliver^2^ (2014) recently reviewed all of this evidence.

Saba^3,4^ and colleagues first pointed out in 2005 that the sudden drop in magnetic flux near the equator is followed, within a few weeks, by a sudden jump in new magnetic flux at 35-degree latitude belts at which the onset of new cycle spots soon follows. Their observations indicate the hidden subsurface existence of the activity cycle at higher than 35-degree latitudes in the form of an extended cycle. More recently the extended solar cycle concept has been put on an even firmer footing in a series of papers by McIntosh and colleagues^5,6^. These papers consolidate the evidence into a picture of ‘activity bands’ that migrate toward the equator from initial appearance at 55-degree latitude or so. Their primary source for evidence of long-lived equatorward-migrating bands are the virtually always present coronal bright points seen in various short wave-length radiation.

**Figure 1:**
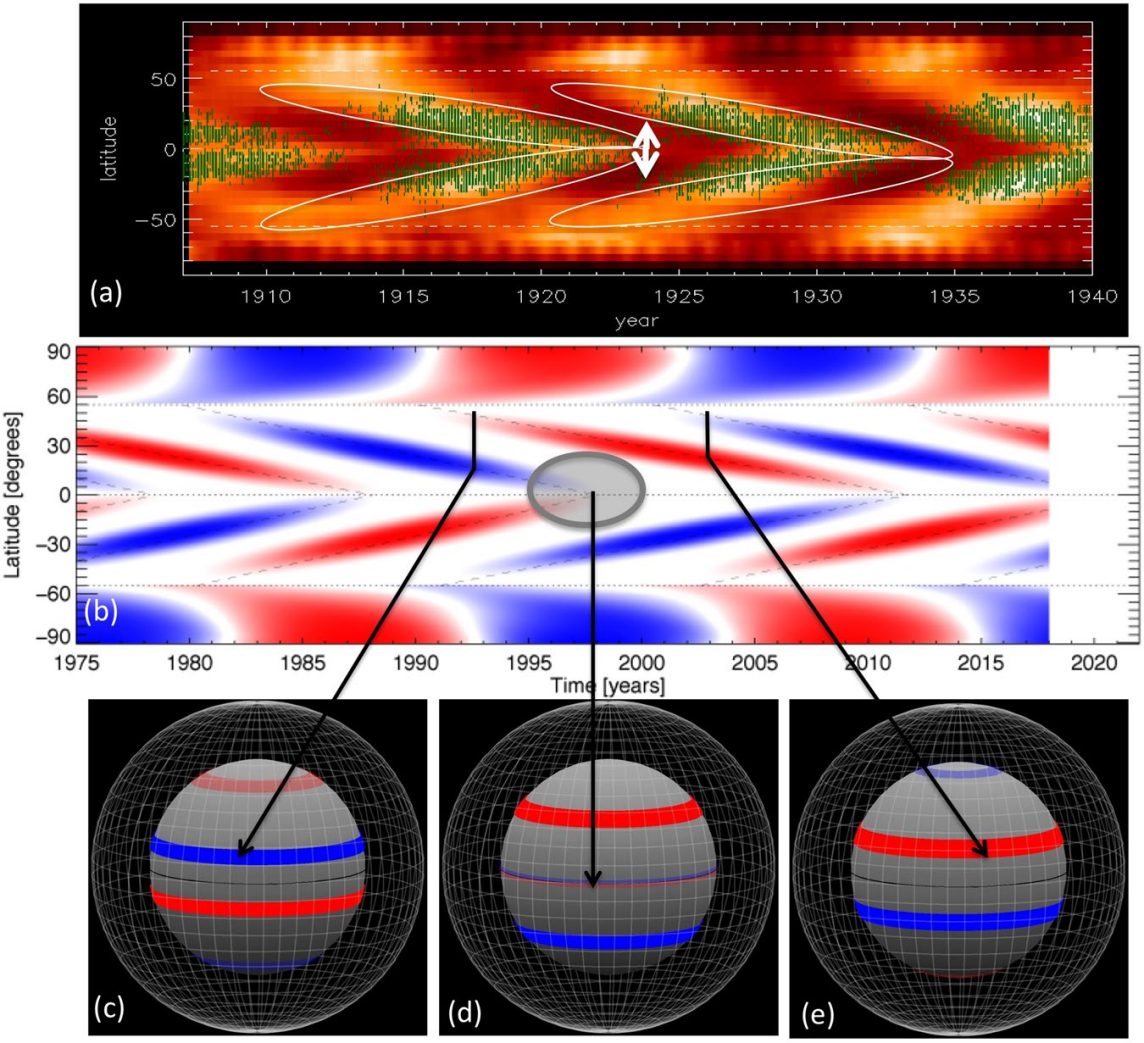
Top frame (**a**) shows observational time-latitude diagram of extended solar cycle in which Ca II K network bright elements are plotted in white-shades, and superimposed on that is the sunspot cycle in green scatter-plot; middle frame (**b**) (regenerated from McIntosh *et al*. 2014) shows data-inspired representation of migration of the activity band during an extended cycle; bottom frames (**c**,**d**,**e**) depict, in each hemisphere, the configuration of oppositely-directed double toroidal magnetic bands in the Sun’s shear-layer (tachocline, denoted by solid gray sphere) during different phases of the extended solar cycle. White mesh-surface in the outer sphere denotes the surface of the Sun. Blue/faint-blue indicates negative (azimuthally opposite to the direction of rotation) and red/faint-red positive (along the direction of rotation).

Figure 1 gives a representation of the extended cycle as observed in Calcium II K network bright points^7,8^ and other observational data such as sunspots, and corresponding magnetic field configurations in the solar tachocline. Frame (a) displays Calcium II K network bright points from Kodaikanal Observatory superimposed on the traditional butterfly diagram of sunspots from Royal Greenwich Observatory (RGO) for 3 cycles early in the twentieth century. White ovals are added to highlight the migration of brightness, clearly showing the time extent of a single cycle as well as the overlap between successive cycles. The cessation of a cycle is denoted by the vertical white arrows. Frame (b) is a data-inspired rendering of the same patterns, regenerated following McIntosh *et al*.^5^, with red and blue shading to separate cycles in North and South hemispheres by magnetic polarity and time. These patterns can be used to roughly infer the location and strength of the underlying deep-seated toroidal fields that are the origin of successive cycles in all the magnetic signatures as schematically represented in the bottom row of Fig. 1. In addition, if we extrapolate the patterns in Fig. 1b forward in time, by analogy with earlier extended cycles, we can estimate that the new sunspots of next cycle will first appear by 2020. There exist some time periods when two toroidal magnetic bands in each hemisphere, one of each polarity, are present in the Sun at the same time, such as in frames (c) and (e). The high-latitude magnetic bands in Fig. 1(c,e) are marked faint, because they are still weak, newly generated next cycle’s magnetic field. Since they did not get enough time to be amplified by the dynamo action yet, they are not buoyant enough to erupt to the surface as coherent sunspots, but they can produce ephemeral regions, which are observed before a new sunspot cycle begins. Frame (d) of Fig. 1 denotes that phase of the cycle when the cessation of old cycle occurs. The disappearance of brightenings near the equator imply the disappearance of underlying toroidal field there; observations^5,6^ indicate that this disappearance gives signal for the appearance of new cycle spots in a short time later, within a few days to weeks.

It is a well-established fact that the spot-producing magnetic fields are generated by the solar dynamo. The so-called flux-transport dynamo models^9,10^ are successful in reproducing the qualitative features of time-latitude diagrams like Fig. 1, in the form of equatorward migration of spot-producing toroidal fields and poleward drift of polar fields. But so far the flux-transport dynamos have been modeled essentially in the kinematic regime and without coupling the dynamics of the tachocline, and therefore lack the capability to send a dynamical signal rapidly from the equatorial regions to mid-latitudes in the solar interior after the old sunspot cycle ceases. Global 3D MHD convectively driven dynamos have also achieved success in this way, so that the solar activity starting at mid-latitudes evolve toward higher and lower latitudes^11^. But the crucial aspects of tachocline dynamics are not correctly captured yet in these models either. Here we intend to answer a more detailed, precise question about possible links between low and mid-latitudes in the solar interior, namely what causes sunspots to suddenly appear where previously there were only bright points and ephemeral regions. At this time the overall level of solar activity suddenly rises.

We demonstrate here that the annihilation of the activity band of the previous cycle by the oppositely directed band near the equator in the opposite hemisphere can trigger a solar “tsunami” that migrates poleward with a gravity wave speed. In this concept a sudden sharp drop in low latitude bright points would be followed by the sudden rise of new cycle bright points, in a time that could be as short as a few weeks. We show that such a short travel time for a magnetic ‘signal’ might be achieved by a poleward propagating gravity wave in the solar tachocline. Plausible gravity wave speeds fit with the observed time difference of the bright point changes. By contrast, a solar dynamo wave itself, by observations, takes a whole sunspot cycle of approximately 11 years to travel the same distance, so the dynamo waves have distinctly different speed and properties compared to tachocline gravity waves. Thus we demonstrate in this paper that most plausibly the solar tsunami is responsible for the sudden appearance of the new cycle’s sunspots. Using an MHD shallow water tachocline model, we simulate the magnetically triggered solar tsunami to estimate how quickly after the annihilation of old cycle’s magnetic bands the new cycle’s sunspots manifest at the surface.

## Concept of an MHD Tachocline Tsunami

Tsunamis are well-known phenomena in the Earth’s oceans. They are strong gravity waves on the surface of the ocean excited usually by earthquakes, volcanic eruptions or major landslides that occur on or below the ocean floor. As such they are excited suddenly, propagate rapidly over great distances, but are singular, isolated events, very unlike the usual ocean waves we have all seen. A somewhat related event is the sudden breaking of a water reservoir dam on land.

**Figure 2:**
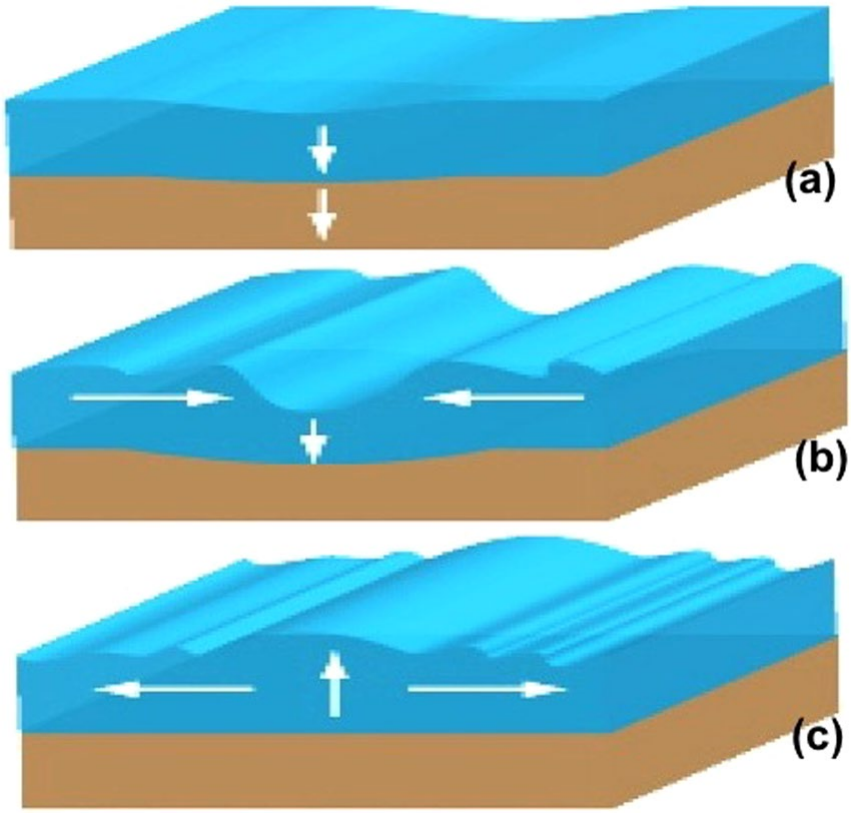
(**a**) Subsidence of Earth’s crust (created by underwater earthquake) causes ocean fluid to fill in to compensate the area (shown by downward white arrows); (**b**) adjacent fluid (marked by two horizontal arrows) from both directions of the epicenter rushes to displaced area; (**c**) surplus of fluid cannot be contained and hence must return back from the epicenter to the adjacent area. By the time the fluid builds power and consequently tsunami is produced.

Figure 2 shows a schematic representation of a tsunami in the ocean caused by a sudden slumping of the sea-bed below (white vertical arrows). The event start is shown in (frame a). The ocean responds by a column of water sinking at the same place. Very quickly water flows in from both sides, while high amplitude gravity waves propagate away from the earthquake location. The inflowing water ‘overshoots’, leading to the ocean bulging upward there (frame b), followed by more outward propagating gravity waves (frame c). The question is: can this kind of event also happen in the Sun?

The sunspot-producing strong toroidal magnetic fields most likely get generated in the tachocline, the Sun’s shear-layer located at/near the base of the convection zone. In a very similar way as described above, MHD tsunamis can be excited in the solar tachocline, and can be responsible for triggering new cycle activity immediately following the cessation of old cycle activity seen at the solar surface. How might this be possible? In recent days the solar tachocline has been most popularly modeled in global MHD shallow-water formalism^12^, a hydrodynamic version of which has been used for understanding oceanic and atmospheric global flows. In the global MHD shallow-water tachocline, the presence of toroidal magnetic field that is in force equilibrium with the tachocline plasma-fluid requires that there be higher gas pressure on the poleward side of the band than on the equatorward side^13^ to push back against the curvature stress from the toroidal magnetic bands. Such a system is in magnetohydrostatic balance in the local vertical direction, so higher polar side pressure must come from additional mass piled up there. Thus the toroidal ring acts a magnetic ‘dam’, holding extra mass on the poleward side. If this dam were to break suddenly, or the whole toroidal ring suddenly disappears, then a solitary gravity wave would propagate initially equatorward, until it encountered some other barrier and reflected back.

How can this MHD dam be broken suddenly? One possibility is that when the late cycle toroidal field bands in North and South hemispheres get close to the equator, they suddenly annihilate each other, being of opposite sign. But at this time there would be usually already a second toroidal band at higher latitudes in both hemispheres, the beginnings of the next cycle of sunspots, as shown in Fig. 2(d) (in the middle of the bottom frame). If the two equatorial bands suddenly cancel each other out, then the mass distribution in the tachocline is no longer in equilibrium in low latitudes. The result is that gravity waves or tsunamis propagate to the equator, but then reflect off each other and propagate poleward. When these waves reach the higher latitude bands in each hemisphere, the local equilibrium of these bands is disrupted, leading to the sudden injection of magnetic flux into the convection zone above, which then rises to the photosphere due to magnetic buoyancy to form new ephemeral regions, bright points and ultimately sunspots.

For equal bands of opposite sign at the same low latitude in each hemisphere there would in principle be total annihilation of toroidal flux right at the equator. But we know that often one hemisphere is ahead of the other in phase. In this case, depending on the low latitude meridional circulation, the earlier band could propagate a short distance into the other hemisphere and annihilate there. Similarly, if one late cycle band is stronger than the other (has more total toroidal flux remaining in it), then the annihilation may not be complete, which could lead to the hemisphere with stronger flux continuing the old cycle, even producing a few very late cycle very low latitude spots.

It has already been established (see, e.g. Gilman and Cally, 2007 and references therein)^14^ that the tachocline differential rotation and toroidal magnetic fields are jointly unstable to disturbances with low longitudinal wavenumbers in the form of Rossby waves^15^. Nonlinear simulations of these Rossby waves in a so-called shallow-water model of the tachocline also indicate the presence of finite amplitude gravity waves (see, e.g. Dikpati 2012)^16^.

The MHD of the qualitative solar tsunami picture we have described here is dominated by axisymmetric structures (longitude wave number m = 0). But in actual simulations, m = 1 and even m = 2 modes also contribute. The annihilation process itself could be longitude-dependent, at least in part. We present here nonlinear global MHD simulations of a shallow water tachocline system to show our postulated physical mechanism can actually occur. Our initial state will be one in which the annihilation has just occurred and the system is suddenly out of equilibrium.

## Model and Results

Following extensive studies of global hydrodynamics (HD) and MHD of the solar tachocline in quasi-3D MHD shallow-water model^12,17–23^, we employ here a global nonlinear MHD shallow-water tachocline model to simulate a solar tsunami.

**Figure 3:**
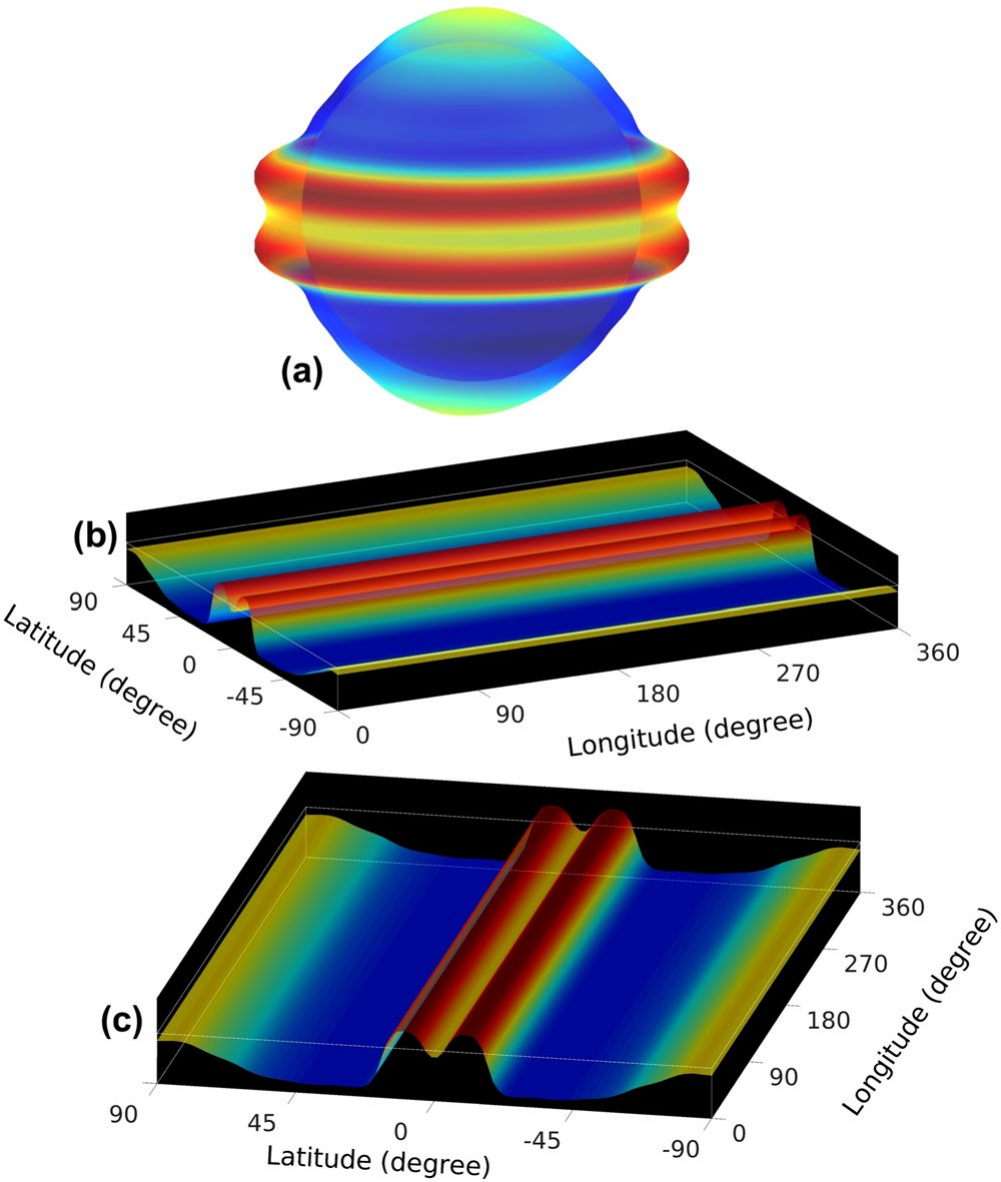
Equilibrium configuration of an MHD shallow-water tachocline top surface (20 times magnified) is presented in 3 perspective views: (**a**) tachocline fluid shell in spherical coordinate; top of gray inner, solid sphere represents bottom of tachocline and outer surface in semi-transparent color-shade the tachocline top-surface (red denotes bulging, blue depression). Two dams (two red bulging) on both sides of the equator arise due to supporting two oppositely-directed strong toroidal magnetic bands of 10-degree width at 5-degree latitudes on both sides of equator (about 150 kGauss peak-field). The two old-cycle’s toroidal bands are not explicitly shown in this picture in order to avoid complexity. (**b**,**c**) Display flattened global tachocline fluid shell respectively viewed along longitude (in panel b) and latitude (in panel c). Tachocline bottom is displayed in black and top-surface in color shades.

Recently such a shallow-water tachocline model has demonstrated that tachocline nonlinear oscillations (TNO), generated by the oscillatory exchange of energies between tachocline differential rotation and magnetized Rossby waves there^15,24^, can successfully simulate the 6–18 months periodicity of the seasonal bursts of solar activity. We initialize the model by a toroidal magnetic configuration as shown schematically in Fig. 1(d), depicting the epoch of cessation of a cycle, during which the old cycle spot-producing toroidal magnetic band has just annihilated with its opposite hemisphere counterpart. The force-balance of the tachocline in this epoch is numerically computed and is presented in Fig. 3 in three different perspective views, namely in spherical polar coordinates, in latitude-longitude planform viewed along longitude, and viewed along latitude.

Two big dams on both sides of the equator in Fig. 3 appear from the plasma fluid that was supporting the strong, spot-producing toroidal bands at the equator until they were annihilated. Assuming that the annihilation of the old cycle’s band is a quick process, the two dams will be prone to create a solar tsunami. Theoretically there should be two more dams due to the presence of two high-latitude toroidal bands, representative of eventually new solar cycle, but these high-latitude dams are a few orders of magnitude weaker than that at the equator and hence almost invisible. In order to focus on the equilibrium structure of plasma fluid of the tachocline, we avoided showing the coexisting double toroidal magnetic bands (a strong band near the equator and a two-orders of magnitude weaker band at high-latitude) in each hemisphere. We first run the model with strong (150 kGauss peak field strength) low-latitude bands of 10-degree width at 5-degree latitude in each hemisphere and two weak (2 kGauss peak) bands at 40-degree latitudes to establish a fully developed solution for the nonlinear evolution of global MHD Rossby waves coupled with differential rotation. For this configuration, the high-latitude toroidal bands govern the dynamics, despite being a few orders of magnitude weaker than the bands at the equator, because they cause the global MHD Rossby wave instability while the system is stable to bands very close to the equator^25,26^.

In the second step of our simulation, we start from this fully developed tachocline dynamics, and make the bands near the equator disappear due to annihilation with their opposite-hemisphere’s counterparts. Assuming that this annihilation is too quick to adjust the equilibrium of the two plasma dams at the equator, the background state will no longer be in force balance, and thus gravity waves will be generated to adjust the dynamics, and consequently trigger a solar tsunami. A sudden change in the global pressure distribution in the system occurs.

**Figure 4:**
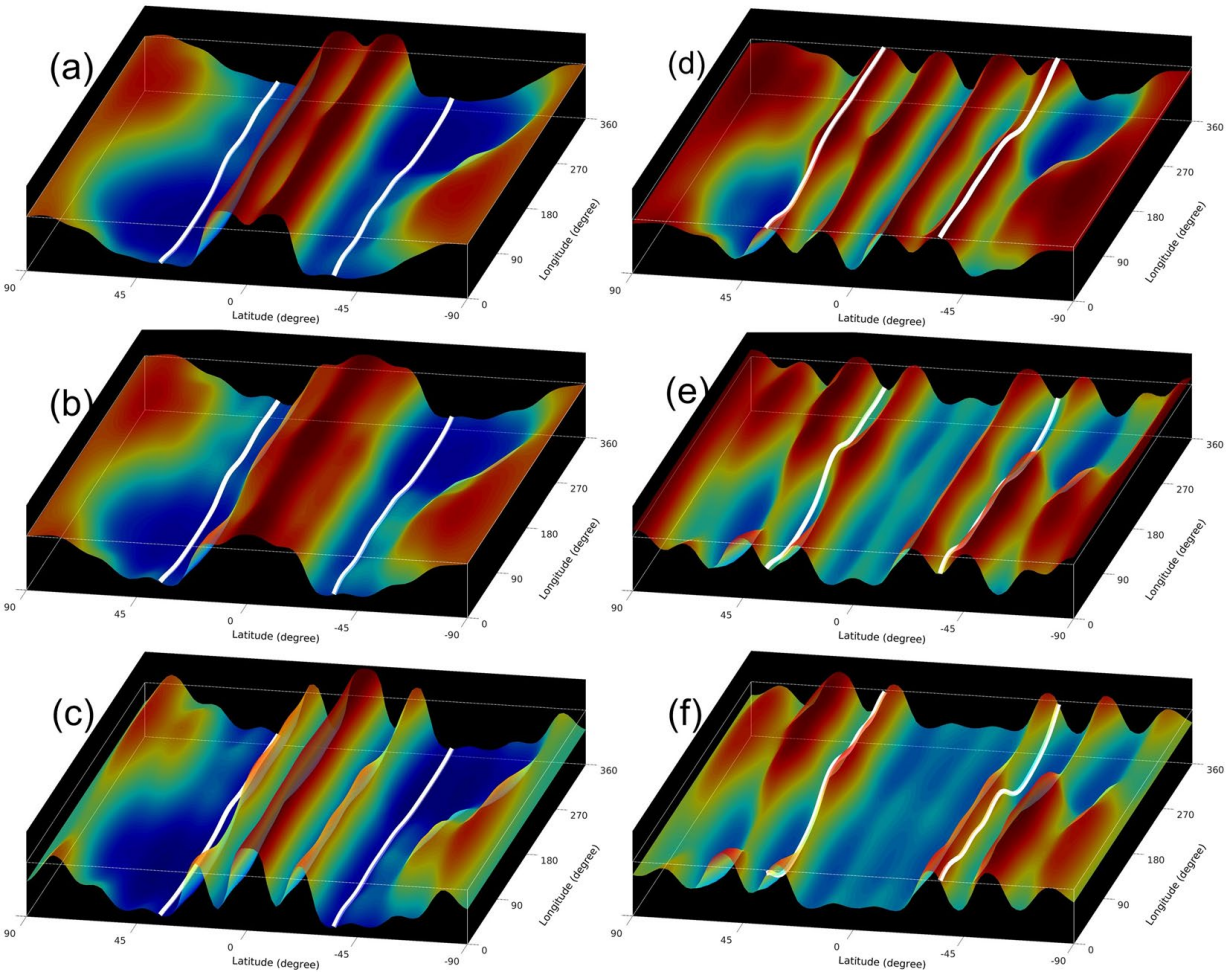
Six snapshots of global tachocline fluid top surface deformations are presented in approximately 4 days interval to show the triggering and development of tsunami, which after reaching the mid-latitudes, lift the weak (nonbuoyant) toroidal bands to help erupt as sunspots at solar surface in approximately a couple of weeks since the cessation of the old cycle.

Figure 4 in six frames shows the progression of the simulation through a whole solar tsunami event. (Frame a) shows the initial state with the two bulges on either side of the equator. The new cycle toroidal band is depicted as a white ribbon in each hemisphere. (Frame b) is early in the evolution, and shows the bulges merging at the equator, followed by reflection off each other (frame c) with the start of poleward propagation. (Frame d) shows the first wave in each hemisphere lifting the toroidal band, (frame e) the first wave propagating poleward of the band, followed by a second wave approaching the band, and (frame f) showing the second wave lifting the toroidal bands again.

Equatorward of the second wave, we also see an essentially flat shallow water surface, highlighting the transient nature of the event. These two episodes of lifting the toroidal bands we infer should set off the injection of new cycle flux into the convection zone that rises to the surface in the form of newly emerging flux, leading in a short time the burst of new cycle spots. We can see that, even though the original gravity waves are mainly m = 0 modes, higher m’s are showing up in the toroidal bands, favoring flux emerging at more than one longitude location. Also, we can see that the whole hemisphere is feeling the poleward propagating gravity waves including near the poles. There are global rearrangements of mass occurring. The changes in surface height are amplified near the poles, because of the convergence of meridians and shortening of high latitude circumference. This is analogous to Earth tsunamis amplifying when they reach shallower coastal waters.

The speed of gravity waves in the solar tachocline is approximately proportional to the square-root of the “effective gravity” (${G}_{eff}$) present there. In fact, the shallow-water gravity wave speed in the solar tachocline, *C*_*g*_, is given by $\sqrt{{G}_{eff}H},$ in which $H$ is the thickness of the tachocline fluid shell. The effective gravity is, in turn, proportional to the percentage the actual (subadiabatic) radial temperature gradient is less than the adiabatic gradient. Near the bottom of the tachocline, this fraction is of order ${10}^{-1}$ (top of the radiative interior) while in the convective overshoot tachocline above, it is more like ${10}^{-5}$. For a gravity wave or tsunami to propagate from the equator to ${40}^{0}$ latitude in about two weeks requires a gravity wave speed of order 300 meteres/second. This is easily achieved in the overshoot tachocline. In fact, the observed time delay between the disappearance of old cycle’s active regions and the first appearance of new cycle’s active regions’ emergence at mid-latitudes can be a measure of what the effective gravity of the tachocline is. We have estimated above, see e.g. in Fig. 1(b), that the next tsunami is due by 2020, and hence within a few weeks from the tsunami new cycle’s sunspots will be born.

## Discussion

It is well-known that in qualitative terms the solar dynamo governs the timing for the generation of the new cycle’s magnetic fields, and hence the approximate onset-timing of a new sunspot-cycle; however the dynamo models do not simulate yet the precise timing of the trigger of new cycle’s sunspots. We have demonstrated here a physical mechanism, the solar tsunami, which gives birth to the new cycle’s sunspots precisely within a few weeks from the cessation of old cycle’s spots. In no ways does this specific tachocline dynamics replace or supersede any operating dynamo process, but instead adds to the precision with which it determines the onset timing. To include our mechanism in a full dynamo requires the inclusion of a realistic solar tachocline self-consistently with associated global MHD processes, something that has not been achieved in any dynamo model yet.

We have simulated how a ‘solar tsunami’ can occur in the solar tachocline by the annihilation near the equator of late cycle toroidal flux in North and South hemispheres. This reconnection event creates a large amplitude gravity wave that propagates poleward in both hemispheres to perturb the high latitude toroidal band of the next sunspot cycle. The example we have given to demonstrate our newly discovered physical mechanism is for bands of equal strengths and latitudinal widths near the equator, oppositely-directed in the North and South hemispheres.

We know that typically a sunspot cycle may not have either the same amplitude or time phase in North and South hemispheres, so we need simulations that are initialized with different low latitude band strengths, and therefore different ‘dam heights’ in the two hemispheres, as well as, perhaps, different low latitude placements. Our preliminary calculations indicate that the weaker dam in a hemisphere produces the tsunami a few days later than the stronger dam in the other hemisphere. In general with different total flux in the two hemispheres there will be only partial annihilation of flux in the stronger hemisphere, so there will remain some damming effect in that hemisphere. In addition, we need to see how the simulated tsunamis change for different effective gravities in the tachocline. The basic gravity wave speed is proportional to the square root of the effective gravity. We will investigate in a forthcoming paper how the results vary with the latitudinal width of both toroidal bands. Narrower bands make dams with steeper front slopes.

The reconnection of equatorial toroidal flux has been considered here to occur instantly, because of global MHD instability deforming the bands, and the fast development of the tsunami over just a few hours. But reconnection may occur over a finite period of time, depending on plasma density. The reconnection can be fast if it is Petschek type^27^, but slow if it is Parker type^28^. However, neither of these theories were designed to apply to the high-density plasma of the solar interior; instead, they apply more to the solar atmosphere where the magnetic field tends to dominate over the inertia of the plasma. We have considered that the reconnection occurs axisymmetrically (longitudinal wave number m = 0), but in reality it might occur at finite m starting at longitudes where toroidal bands in opposite hemispheres, warped by the global MHD Rossby waves, are closest together in latitude across the equator This could be simulated by considering a dam whose latitude itself has longitude dependence in it.

Quite separate from all the future simulation studies described above, the details of how the perturbed high latitude toroidal band of the new sunspot cycle injects toroidal flux into the convection zone should also be modelled. This effort would require a completely different model than the MHD shallow-water system that we have used here. Finally, more study is needed of the observational ‘signatures’ of the triggering of a new cycle seen at the photosphere and above by the equatorial reconnection of the remaining toroidal flux of the previous cycle.

## Methods

To simulate the tsunami in the solar tachocline, we employed the nonlinear, global magnetohydrodynamic shallow-water equation-set (see, e.g. Dikpati *et al*.^24,26^) in a rotating frame (rotating with the solar core-rotation rate ${\omega }_{c}$) and solved them by implementing a pseudo-spectral algorithm for the tachocline plasma-fluid shell in a spherical polar coordinate. Generally in shallow-water approximation, the radial divergence and the variation in density in the fluid-shell are negligible in the equations of motion and mass-conservation. Latitudinal and longitudinal velocities and magnetic fields depend on latitude, longitude and time, but independent of radius; the radial components of velocity and magnetic field vary linearly with height. From helioseismic measurements the solar tachocline is known to be very thin (~3–4% of the solar radius at tachocline depth), therefore a shallow-water model can be applied for tachocline plasma-fluid shell.

Simulations of tsunami in tachocline have been performed for selected model parameters, which are the “effective gravity” (${G}_{eff}$) of the plasma-fluid shell, latitude-location, width and strength of two oppositely-directed banded toroidal magnetic field, and the amplitude of tachocline latitudinal differential rotation ($\omega$, given by $\omega$= ${s}_{0}-{s}_{2}{\mu }^{2}-{s}_{4}{\mu }^{4}-{\omega }_{c}$, see, e.g. Dikpati *et al*. 2018 for details^24^). Here ${s}_{0},\,{s}_{2},\,{s}_{4}$ denote the parameters for determining the pole-to-equator differential rotation amplitude (s, which is $({s}_{2}+{s}_{4})/{s}_{0}$). $\mu$ denotes the sine latitude. The non-dimensional effective gravity (${G}_{eff}$) can be written, using dimensional gravity (${g}_{actual}$) and subadiabatic stratification ($\delta$) at the tachocline depth (${r}_{0}$), as ${G}_{eff}=\frac{\delta \times \,{g}_{actual}\,\times \,H}{{({r}_{0}\times {\omega }_{c})}^{2}}$. As mentioned earlier, $H$ denotes the reference-state (unperturbed) thickness of the tachocline fluid shell and ${\omega }_{c}$ the Sun’s core-rotation rate. ${{G}}_{eff}$ is ${G}_{actual}\,\times \,\delta$, and $\delta$ varies from approximately ${10}^{-1}$ to ${10}^{-5}$ from the bottom (radiative part) to the top (convective overshooting part) of the tachocline. This means that effective gravity in the tachocline varies from about 100 to 0.01.

Nonlinear shallow-water code for simulating tachocline dynamics is written in dimensionless units with ${r}_{0}$ denoting the dimensionless length and 1/${\omega }_{c}$ the dimensionless time. Therefore, unit dimensionless time is approximately 3.65 days.

To mimic an extended cycle’s magnetic field configuration, we prescribe oppositely directed toroidal magnetic bands in each hemisphere by using Gaussian profile in latitude (see e.g. Dikpati & Gilman 1999 and Cally *et al*. 2003);^29,30^ the Gaussian’s peak and full-width at half-maximum respectively determine the toroidal magnetic band’s strength and width. Tsunami simulation is initiated here from a starting configuration of a pair of oppositely-directed old cycle’s toroidal bands (150 kGauss peak field-strength and 10-degree width), each placed at 5-degree latitudes of north and south hemispheres, along with another pair of weak (2 kGauss), opposite-polarity new cycle’s bands of 10-dgree width again at mid-latitudes (40-degrees) in north and south hemispheres.

## Data Availability

Solar tsunami simulation-outputs are obtained as spherical harmonic coefficients, which are stored in the High Performance Storage System of NCAR’s supercomputing center as well as in HAO’s network attached storage system. Three-dimensional data representing the tsunami variables and corresponding graphics and movies can be derived from stored spherical harmonics. Kodaikanal observatory data and all model-outputs including model and graphics codes will be available upon request, respectively from Dipankar Banerjee and Mausumi Dikpati.
